# Hypoxia upregulate TPM4 expression to strengthen epithelial-mesenchymal transition that promotes lymph node metastasis of papillary thyroid cancer

**DOI:** 10.7150/jca.116524

**Published:** 2025-07-11

**Authors:** Zhufeng Li, Fuxin Li, Xunzhen Jiang, Yingxi Li, Lanning Jia, Xiaoning Wang, Yuanchao Liu, Ruoyu Jiang, Ke Zhao, Guangmei Pu, Yao Tian, Yizeng Wang, Xianghui He

**Affiliations:** 1 Department of General Surgery, Tianjin Key Laboratory of Precise Vascular Reconstruction and Organ Function Repair, Tianjin Medical University General Hospital, Tianjin General Surgery Institute, Tianjin, 300052, China.; 2 Department of General Surgery, Tianjin Haihe Hospital, Tianjin, 300350, China.; 3 Immunology Department, Key Laboratory of Immune Microenvironment and Disease (Ministry of Education), Tianjin Medical University, 300070, Tianjin, China.; 4 Department of Molecular Pharmacology, Tianjin Medical University Cancer Institute and Hospital, National Clinical Research Center for Cancer, Key Laboratory of Cancer Prevention and Therapy, Tianjin, Tianjin's Clinical Research Center for Cancer, Tianjin, 300060, China.

**Keywords:** papillary thyroid cancer, hypoxia, TPM4, lymph node metastasis, epithelial-mesenchymal transition

## Abstract

Papillary thyroid cancer (PTC) exhibits a high propensity for lymph node metastasis (LNM), significantly impacting postoperative recurrence and patient prognosis. The hypoxic microenvironment critically drives tumor progression by promoting PTC dedifferentiation. Through integrated bioinformatics analysis combining weighted gene co-expression network analysis and machine learning approaches on TCGA data, we identified TPM4 as a key hypoxia-responsive gene in PTC and validated its association with LNM using GEO datasets. Gene set enrichment analysis demonstrated that patients with high TPM4 expression in both TCGA and GEO databases showed significant enrichment in hypoxia and epithelial-mesenchymal transition (EMT) pathways. Single-cell pseudotime analysis revealed concurrent increases in hypoxia pathway enrichment, TPM4 expression, and EMT pathway activation during cell differentiation. Experimental validation using RT-qPCR and Western blot analyses confirmed that hypoxia-induced TPM4 upregulation activated EMT signaling. Functional assays demonstrated that TPM4 enhanced cellular invasion and migration capabilities. Our findings illuminate a novel mechanism whereby the hypoxic tumor microenvironment promotes lymph node metastasis in PTC through TPM4-mediated activation of EMT signaling, providing new insights into LNM of PTC.

## Introduction

Thyroid cancer (TC) incidence has increased globally in recent years^1^, with papillary thyroid carcinoma (PTC) representing over 90% of differentiated thyroid cancer (DTC) cases. While PTC frequently presents with early lymph node metastasis (LNM), there remains a lack of specific diagnostic markers^2^. Despite advances in preoperative screening, occult cervical LNM occurs in up to 51% of PTC patients without apparent LNM^3^. Furthermore, the number of lateral cervical LNM correlates with local recurrence rates, even though PTC generally has a favorable prognosis^4^. Therefore, identifying novel biomarkers for accurate LNM prediction is crucial for improving patient stratification and personalizing treatment strategies.

Differentiated thyroid cancer frequently exhibits downregulation or loss of iodine metabolism-related genes, leading to dedifferentiation, accelerated tumor growth, increased LNM propensity, reduced ^131^I uptake, and poor prognosis^5^, ^6^. This dedifferentiation process involves multiple pathways, including gene mutations, epigenetic modifications, autophagy, and tumor microenvironment alterations^5^, ^7^, ^8^. For instance, hypoxia-inducible factor 1α modulates NIS localization through β-catenin, inhibiting iodine uptake in thyroid cancer cells^9^, ^10^. Additionally, clinical studies have demonstrated that autophagy activity correlates with thyroid cancer differentiation, with reduced autophagy potentially contributing to TC dedifferentiation and ^131^I treatment resistance^11^, ^12^. These findings collectively underscore the critical roles of hypoxia and autophagy in driving PTC dedifferentiation.

The progression from differentiated to dedifferentiated states in thyroid cancer often involves Epithelial-Mesenchymal Transition (EMT), a crucial process in tumor initiation, progression, metastasis, and drug resistance. Multiple signaling pathways, including TGF-β and Notch, serve as key regulators of EMT^13^, ^14^, and their inhibition has shown promise in preventing tumor metastasis and recurrence. In this context, understanding the molecular mechanisms driving EMT becomes essential for developing targeted therapeutic strategies. Tropomyosin 4 (TPM4), a non-muscle tropomyosin essential for cytoskeletal maintenance^15^, has garnered increasing attention due to its overexpression in various malignancies^16^-^18^. Given that cell migration depends on coordinated actin cytoskeleton organization^19^, understanding TPM4 expression in PTC with LNM may provide valuable insights into metastatic mechanisms.

To address the critical knowledge gap regarding the molecular mechanisms of PTC progression and metastasis, we investigated the relationship between hypoxia, autophagy, and TPM4 expression by developing hypoxia and autophagy phenotype scores. Our study represents the first comprehensive analysis of TPM4 role in TC, particularly in the context of LNM. Using an integrated approach combining TCGA and GEO databases with single-cell sequencing analysis and experimental validation, we aimed to elucidate the potential of TPM4 as a novel biomarker for PTC progression and metastasis. This work not only advances our understanding of the molecular mechanisms underlying thyroid cancer progression but also provides new insights for potential therapeutic strategies.

## Materials and methods

### Dataset collection

We compiled a comprehensive dataset comprising 497 papillary thyroid carcinoma (PTC) cases from The Cancer Genome Atlas (TCGA) database (https://portal.gdc.cancer.gov/) for primary analysis. This dataset excludes 7 cases with pathology-confirmed non-PTC diagnoses and 8 metastatic samples. For validation, we integrated 112 PTC cases from five independent Gene Expression Omnibus (GEO) datasets (http://www.ncbi.nlm.nih.gov/geo). The merged GEO cohort consisted of samples from five studies: GSE129562 (8 cases), GSE153659 (24 cases), GSE29625 (20 cases), GSE33630 (27 cases), and GSE60542 (33 cases). Notably, we excluded 22 radiation-exposed cases from the GSE33630 dataset. Raw GEO data underwent preprocessing and normalization using the R packages 'sva' and 'limma'. We obtained TCGA RNA-seq data for PTC cases with comprehensive clinical annotations, with all GEO datasets providing detailed LNM information.

### Single sample gene set enrichment analysis (ssGSEA)

We extracted 81 hypoxia-related genes from the HARRIS_HYPOXIA gene set and 158 autophagy-related genes from the REACTOME_AUTOPHAGY gene set in the GSEA database (https://www.gsea-msigdb.org/gsea/index.jsp) (Table S1). ssGSEA was performed across all samples to calculate hypoxia and autophagy phenotype scores for each specimen (Table S2)^20^.

### Weighted gene co-expression network analysis (WGCNA)

Based on ssGSEA scoring results^21^, we categorized TCGA-THCA samples into hypoxia and autophagy phenotypes to establish module-trait relationships. Using the 'WGCNA' R package, we identified modules most strongly associated with the hypoxia phenotype. The module exhibiting the strongest correlation with hypoxia was designated as the key module associated with thyroid cancer dedifferentiation. We defined gene significance (GS) as the correlation between gene expression and individual traits, while module membership (MM) represented the correlation between gene expression and module eigengenes. Hub genes within the key module were identified using the criteria of GS > 0.2 and MM > 0.8.

### Least absolute shrinkage and selection operator (LASSO) regression analysis

We performed LASSO regression analysis using the 'glmnet' R package to identify target genes^22^. The optimal lambda value was selected to minimize cross-validation error.

### Functional enrichment analysis

We conducted comprehensive functional enrichment analysis of the identified hub genes using the 'clusterProfiler' R package^23^, ^24^. The analysis included Gene Ontology (GO) terms—covering biological process (BP), molecular function (MF), and cellular component (CC)—as well as Kyoto Encyclopedia of Genes and Genomes (KEGG) pathways. Additionally, Gene Set Variation Analysis (GSVA) was employed to assess signaling pathway differences among patients^25^. Enrichment results were considered significant at FDR q-val < 0.05.

### Screening of key genes based on multiple machine learning (ML) methods

We implemented a multi-model machine learning approach to identify key genes associated with hypoxia in PTC. This approach incorporated four distinct algorithms: extreme gradient boosting (XGB), random forest (RF), generalized linear model (GLM), and support vector machine (SVM)^26^-^29^. The 'DALEX' R package was utilized to illustrate the distribution of model residuals across the four ML methods. Model performance was evaluated using the 'DALEX' R package to visualize residual distributions across all four methods. Additionally, we assessed model discrimination using Receiver Operating Characteristic (ROC) curve analysis and Area Under the Curve (AUC) calculations via the 'pROC' R package. The root mean square error (RMSE) loss after permutations is used to assess feature importance. The greater the increase in RMSE loss after permuting a feature, the greater its impact on the model, indicating that the feature is more important. Key genes were determined by identifying the overlap among the top five genes from each machine learning model.

### Application of HPA and GEPIA online databases

We utilized the Human Protein Atlas (HPA, https://www.proteinatlas.org/) to examine the immunohistochemical staining patterns of target genes in normal thyroid and PTC tissue. The Gene Expression Profiling Interactive Analysis 2 (GEPIA 2, http://gepia2.cancer-pku.cn/) platform was employed to analyze correlations between target genes and the hallmark hypoxia gene set.

### Gene set enrichment analysis (GSEA)

To investigate biological functions associated with elevated TPM4 expression, we performed GSEA comparing high- and low-TPM4 expression groups in both TCGA-THCA and merged GEO cohorts. Analysis was conducted using GSEA software (version 4.2.3) with the h.all.v2024.1.Hs.symbols.gmt gene set. Results were considered statistically significant at p < 0.05.

### Single-cell RNA sequencing analysis

We analyzed single-cell RNA sequencing (scRNA-seq) data from PTC with LNM, partially sourced from the GEO database (accession number: GSE184362). The dataset comprised nine samples from four patients (P02, P03, P05, P10), including matched primary tumors and metastatic lymph nodes, plus one adjacent normal tissue sample (P03). These were subsequently redesignated as P01-P04.

Quality control was performed using the 'Seurat' R package. We excluded cells that met any of the following criteria: detected genes < 200 or > 2,500, total unique molecular identifiers (UMI) < 500, or mitochondrial gene expression > 10%. Doublet cells were identified through the intersection of results from both Scrublet and DoubletFinder algorithms and subsequently removed. Copy number variations (CNVs) in epithelial cells were computed from the scRNA-seq data using the 'infercnv' R package^30^.

Cluster-specific marker genes were identified using the FindAllMarkers function, while differential gene expression analysis was conducted using the FindMarkers function. Cell lineage annotation was performed by integrating results from the 'SingleR' R package and the Annotation of Cell Types (ACT, http://xteam.xbio.top/ACT) database. Data visualization was performed using both t-Distributed Stochastic Neighbor Embedding (tSNE) and Uniform Manifold Approximation and Projection (UMAP) algorithms. Differentially expressed genes (DEGs) were filtered using thresholds of adjusted p-value < 0.01 and |log2FC| > 0.585. Cell differentiation trajectories were constructed and visualized using the 'monocle' R package^31^.

### Cell line culture

Human embryonic kidney 293T (HEK-293T) cells and the PTC-derived cell line K1 were maintained in high-glucose Dulbecco's modified Eagle medium (H-DMEM, Bdbio, China) supplemented with 10% fetal bovine serum (FBS, Bdbio, China) and 1% penicillin/streptomycin. The PTC-derived TPC-1 cell line was cultured in Roswell Park Memorial Institute-1640 (RPMI-1640, Bdbio, China) with identical supplements. All cells were maintained at 37°C in a humidified atmosphere containing 5% CO2.

### Chemical hypoxia induction

Chemical hypoxia was induced using cobalt chloride (CoCl_2_, Sigma-Aldrich), which stabilizes HIF-1α by preventing its degradation^32^. K1 and TPC-1 cells were cultured to approximately 80% confluence before treatment with CoCl_2_ at concentrations of 0.1, 0.2, or 0.4 mM for 24 hours.

### Lentiviral construction and cell transfection

TPM4-targeting shRNAs and overexpression plasmid (oe-TPM4) were designed and synthesized by Synbio Technologies. A non-targeting shRNA vector (sh-NC) served as the control (pLenti-U6-CBH-TurboGFP-P2A-Puro-ncshRNA). Sequence information is provided in Supplementary Table S3.

For lentivirus production, target plasmids were co-transfected with packaging plasmids (pPAX8 and pVSVG) into HEK-293T cells using Lipofectamine 3000 (Invitrogen). Viral supernatant was harvested 48 hours post-transfection, cleared by centrifugation (4,000g, 10 minutes, 4°C), and stored at -80°C. K1 and TPC-1 cells were seeded at 1.0 × 10^6^ cells per well in 6-well plates and transduced with lentivirus 24 hours later. Transduced K1 and Transduced TPC-1 cells underwent puromycin selection after an additional 24-hour incubation.

### Real-time quantitative polymerase chain reaction (RT-qPCR) analysis

Total RNA was extracted using TRIzol reagent (Vazyme Biotech Co., Ltd, China) and reverse-transcribed using the RevertAid First Strand cDNA Synthesis Kit (Vazyme Biotech Co., Ltd, China). RT-qPCR was performed using SYBR Green Master Mix (Vazyme Biotech Co., Ltd, China) according to the manufacturer's protocol. Primer sequences were listed in Supplementary Table S3.

### Wound healing assay

Cells were seeded in 6-well plates (1.0 × 10^5^ cells/well) and cultured to confluence. After switching to DMEM containing 1% FBS, a scratch wound was created using a 10-μL pipette tip. Wound closure was monitored at 0 and 24 hours. The imaging for the scratch assay conducted on shTPM4-TPC1 and shTPM4-K1 cell lines expressing GFP plasmid were performed using a blue light source with an excitation wavelength of approximately 488 nm.

### Transwell migration assay

Cell migration was assessed using 8-μm pore transwell chambers (Corning Incorporated, USA). Cells were trypsinized and resuspended in serum-free medium, with 4×10^4^ cells seeded in the upper chamber. The lower chamber contained complete medium with 10% FBS. After 24-hour incubation, non-migrated cells were removed, and migrated cells were fixed with methanol, stained with crystal violet, and photographed under a microscope.

### Western blotting

Protein lysates were prepared using RIPA buffer containing protease and phosphatase inhibitors. Proteins were separated by electrophoresis, transferred to membranes, and blocked before overnight incubation with primary antibodies at 4°C. After 2-hour incubation with secondary antibodies at room temperature, protein bands were visualized using ECL detection and documented using a chemiluminescent imaging system.

The primary antibodies used included anti-TPM4 (Proteintech, China), anti-HIF1α (Proteintech, China), N-cadherin (Proteintech, China), Vimentin (Proteintech, China), Snail (Proteintech, China), and β-actin (Proteintech, China).

### Clinical specimens and data collection

This study was approved by the Ethical Committee of Tianjin Medical University General Hospital (IRB2024-YX-595-01). We collected surgical specimens and clinical data from 43 PTC patients who underwent curative resection between January and February 2025. Inclusion criteria: Patients who underwent curative resection with pathological confirmation of PTC after surgery. Exclusion criteria: Patients who underwent reoperation or had an incomplete resection. Written informed consent was obtained from all participants in accordance with the Declaration of Helsinki.

### Statistical analysis

Statistical analyses were performed using R (version 4.4.1) and GraphPad Prism (version 9.0). Relationships between variables were evaluated using Spearman correlation analysis. Continuous variables were compared using Student's t-test, while categorical variables were analyzed using chi-square tests. Statistical significance was defined as p < 0.05.

## Results

### Identifying of hypoxia and autophagy phenotype scores in TCGA-THCA dataset

**Figure 1:**
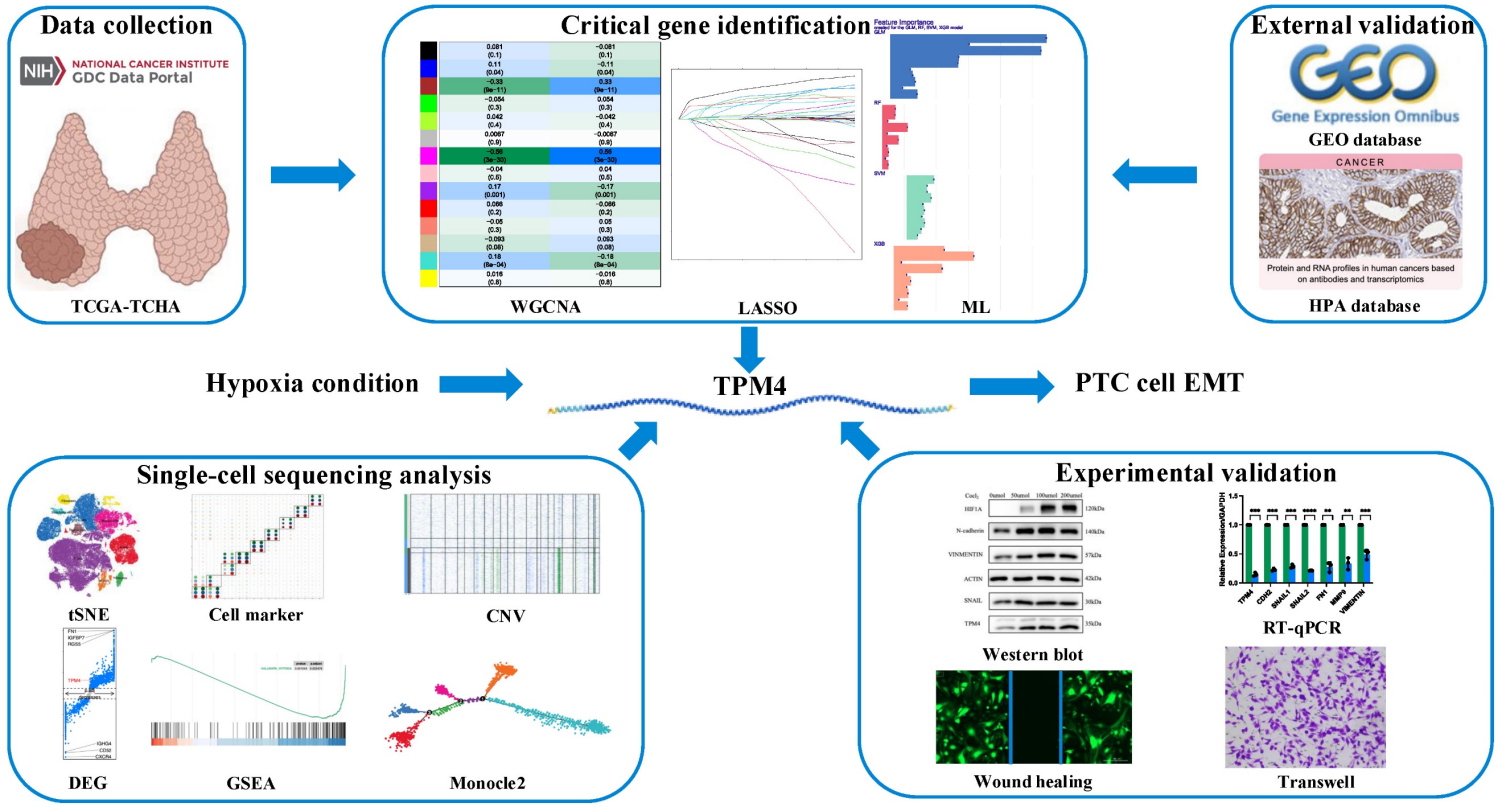
Schematic illustration of the experimental design and workflow of the study.

**Figure 2:**
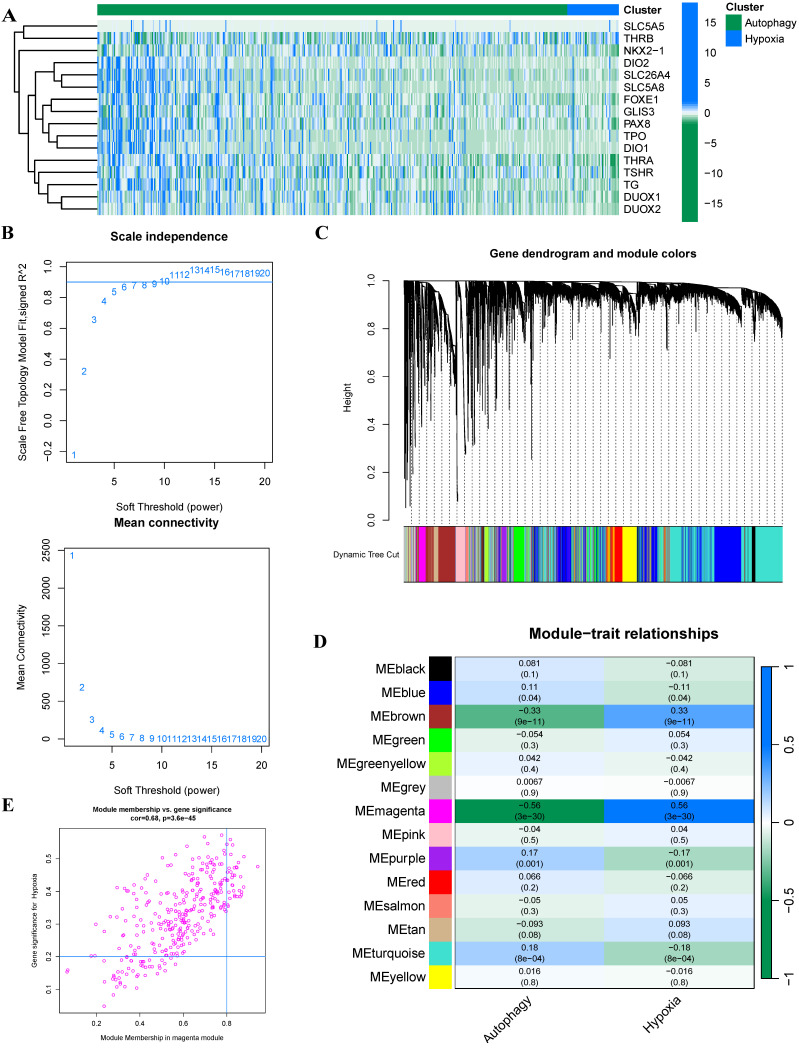
** Construction of phenotype scores and weighted Gene Co-expression Networks (A)** Heatmap illustrating TDS between the two phenotypes. **(B)** Selection of optimal soft-threshold power. **(C)** Gene dendrogram with corresponding module color assignments. **(D)** Fourteen distinct modules identified through WGCNA. **(E)** Systematic selection process of hub genes.

Previous studies had demonstrated that hypoxia and autophagy play crucial roles in PTC dedifferentiation, where hypoxia accelerated PTC dedifferentiation while autophagy served as a protective factor against it. Figure 1 presents a schematic illustration of our experimental design and overall workflow. To investigate the genes promoting PTC progression, we employed the ssGSEA algorithm to establish hypoxia and autophagy phenotype scores using the TCGA-THCA dataset. The heatmap of thyroid differentiation score (TDS) between these two phenotypes revealed that the hypoxic phenotype was significantly associated with PTC dedifferentiation (Figure 2A). We then performed WGCNA analysis incorporating both phenotype scores. The analysis yielded a correlation coefficient exceeding 0.9 (with a soft threshold power of 6), indicating robust correlation suitable for gene module construction (Figure 2B). Through the dynamic tree cutting algorithm, we identified 14 distinct modules (Figure 2C), among which the magenta module exhibited the strongest correlation with the hypoxic phenotype (|R| = 0.56, p = 3 × 10^-30^) (Figure 2D). Subsequently, hub genes within the magenta module were identified as dedifferentiation-related genes, showing significant correlation (cor = 0.68, p = 3.6 × 10^-45^) (Figure 2E).

### Conducting LASSO regression analysis and gene enrichment analysis

**Figure 3:**
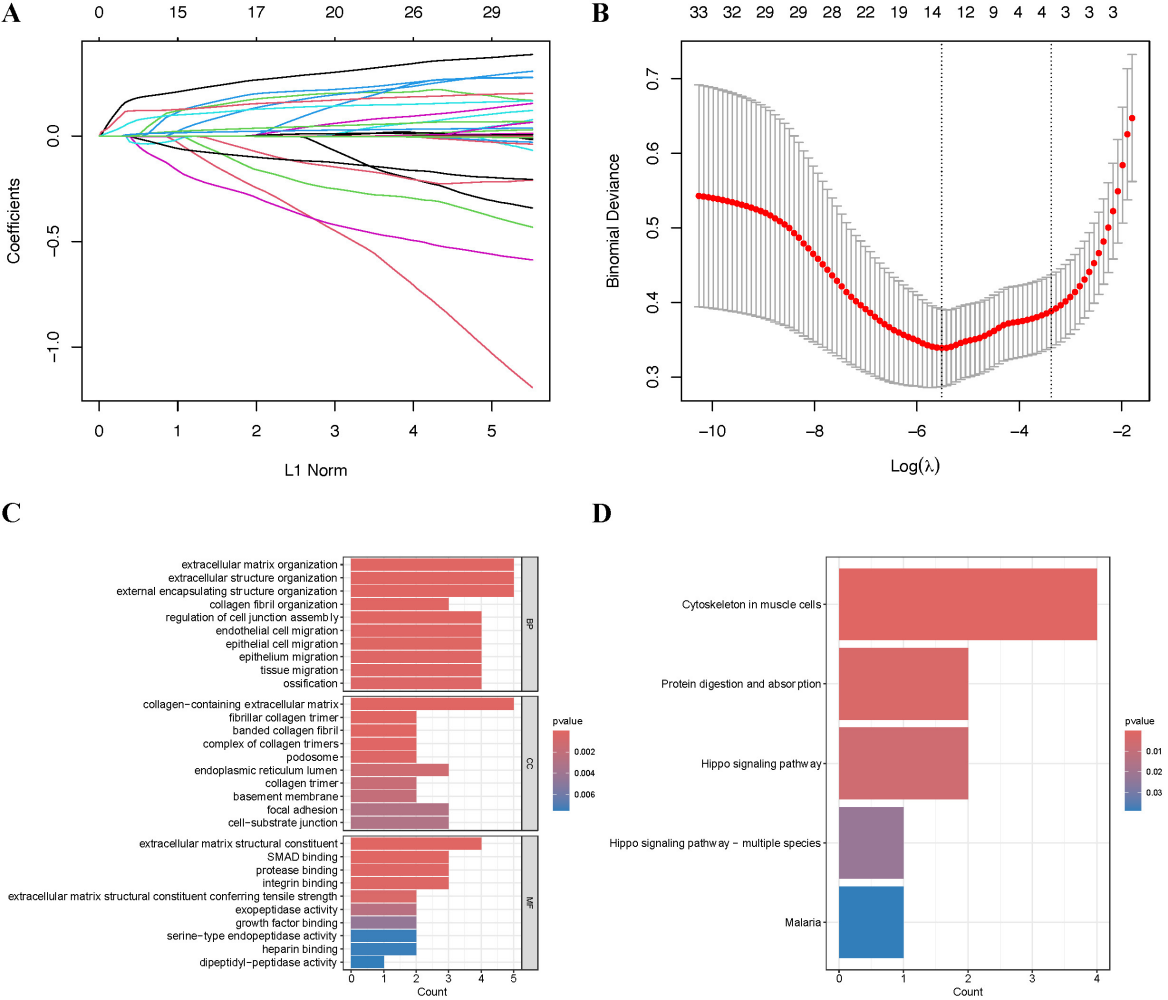
Screening and functional enrichment of hub Genes **(A-B)** Identification of fourteen candidate genes through LASSO regression analysis. **(C)** GO functional enrichment analysis of the 14 genes presented as a bar plot. **(D)** KEGG pathway enrichment analysis of the 14 genes presented as a bar plot.

To mitigate potential overfitting of the hub genes, we implemented LASSO regression analysis to identify key genes with the strongest predictive capacity for PTC progression (Figure 3A-B). This analysis led to the identification of 14 critical genes from the initial pool of 33 hub genes: SRPX2, FRMD6, SPON2, FAP, SNAI2, SH3PXD2A, LOXL1, THBS2, TPM4, PMEPA1, FSTL1, COL5A2, MMP14, and COL3A1. To elucidate the functional roles of these genes, we conducted comprehensive GO and KEGG enrichment analyses. The GO analysis reealed significant enrichment in multiple biological processes, with notable emphasis on epithelial cell migration (BP), cell-substrate junction (CC), and extracellular matrix structural constituent (MF), among other pathways (Figure 3C). Furthermore, KEGG pathway analysis highlighted the cytoskeleton in muscle cells as the most significantly enriched pathway (Figure 3D).

### Screening key genes related to hypoxia in PTC through ML algorithms

**Figure 4:**
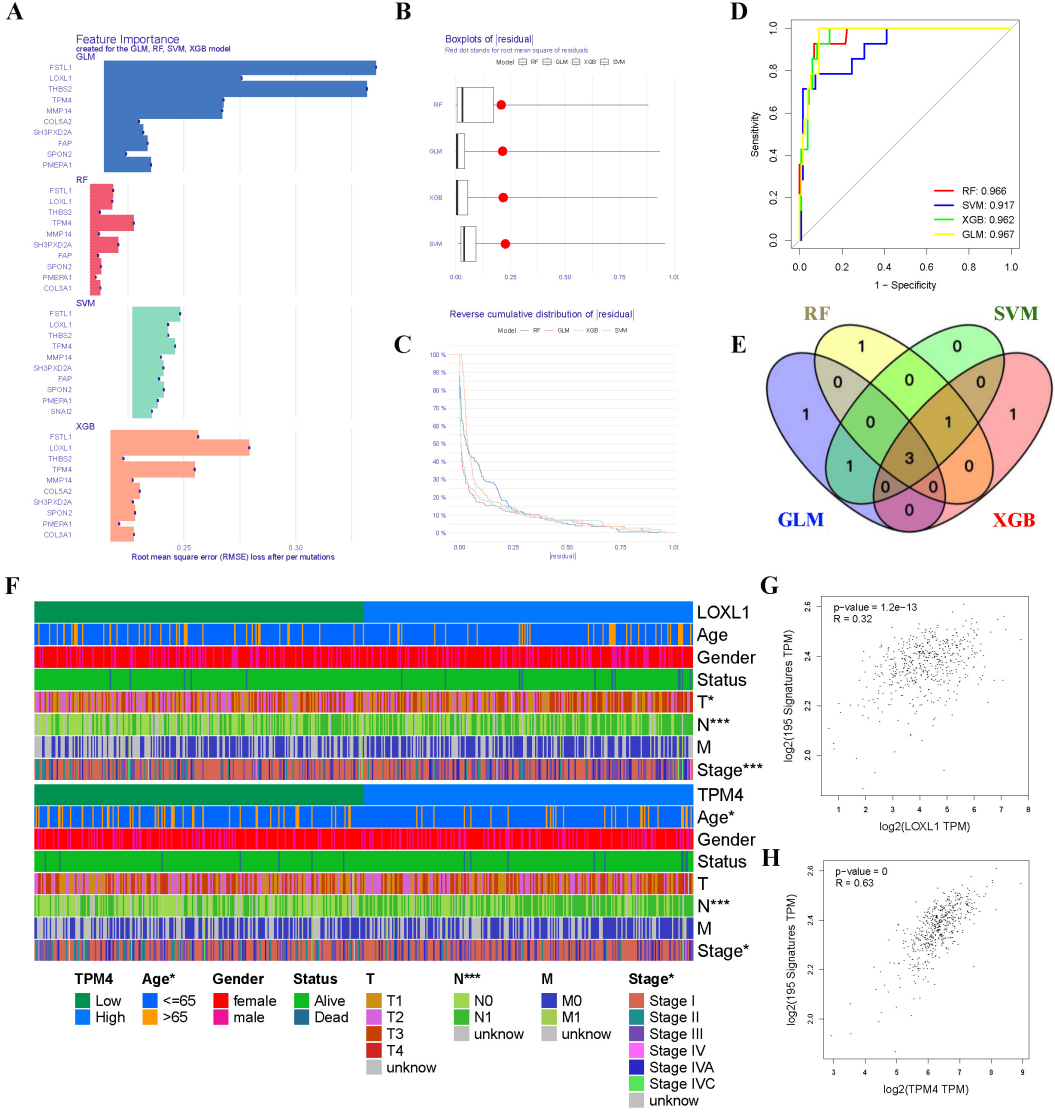
** Machine learning approaches for identifying hypoxia-responsive genes in PTC (A)** Feature importance scores of genes across GLM, RF, SVM, and XGB models. **(B)** Residual distribution boxplots, with the red dot indicating the root mean square of residuals. **(C)** Reverse cumulative distribution of residuals. **(D)** ROC curves demonstrating the predictive performance of GLM, RF, SVM, and XGB models. **(E)** Venn diagram highlighting three conserved genes consistently identified across all four machine learning algorithms. **(F)** Heatmap depicting gene expression patterns and clinicopathological characteristics. **(G-H)** Scatter plots showing correlations between gene expression levels and hypoxia-related gene set expression.

Given the critical role of the hypoxic microenvironment in tumor initiation, progression, and metastasis, we focused on identifying hypoxia-responsive genes in PTC. Four machine learning algorithms (GLM, SVM, RF, and XGB) were employed to prioritize the 14 genes based on their significance as variable indicators (Figure 4A). Among these, the GLM model demonstrated superior performance with the smallest sample residual (Figure 4B-C). The robustness of all four models was evaluated using ROC curve analysis, which revealed excellent predictive capabilities with high AUC values (AUC_GLM_ = 0.967, AUC_RF_ = 0.966, AUC_XGB_ = 0.962, AUC_SVM_ = 0.917) (Figure 4D). Through cross-comparison of the top 5 important genes identified by each ML algorithm, we identified three key genes (TPM4, LOXL1, and FSTL1) at their intersection (Figure 4E). We then generated heatmaps using the TCGA-THCA dataset to visualize the relationships between these three key genes and PTC clinicopathological features (Figure 4F, Figure S1 and Table 1). Our analysis revealed that TPM4 and LOXL1 exhibited strong associations with lymph node metastasis and tumor size in PTC. Furthermore, the expression levels of both TPM4 and LOXL1 showed significant correlation with the expression patterns of hypoxia-related gene set (Figure 4G-H).

### External validation using GEO database and HPA database

**Figure 5:**
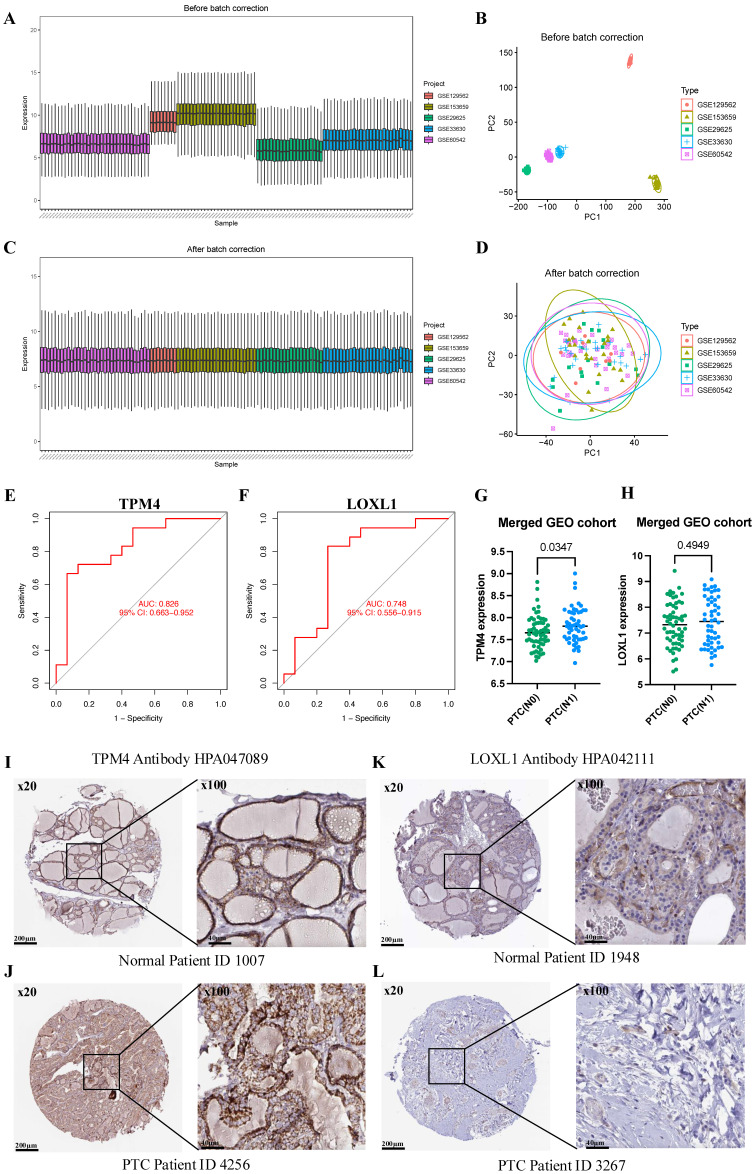
** Evaluating the value of TPM4 for lymph node metastasis in PTC (A-D)** Batch correction of GSE129562, GSE153659, GSE29625, GSE33630, and GSE60542. **(E-F)** TPM4 and LOXL1 in the merged GEO cohort were analyzed using ROC curves. **(G-H)** Differential expression analysis of TPM4 and LOXL1 between PTC with lymph node metastasis (N1) and without lymph node metastasis (N0) groups. **(I-L)** Immunohistochemical staining ofTPM4 and LOXL1 in HPA database.

To comprehensively evaluate TPM4 and LOXL1 as biomarkers for LNM in PTC, we conducted ROC analyses using a merged GEO cohort. We integrated five GEO datasets (GSE129562, GSE153659, GSE29625, GSE33630, and GSE60542) containing LNM clinical information, applying batch correction to ensure data compatibility (Figure 5A-D). The analysis revealed that TPM4 demonstrated superior diagnostic performance with an AUC of 0.826, while LOXL1 showed moderate predictive capability with an AUC of 0.748 (Figure 5E-F). Further investigation of the merged GEO cohort showed significantly elevated TPM4 expression in the PTC lymph node-positive (N1) group compared to the lymph node-negative (N0) group (p = 0.0347). In contrast, LOXL1 expression showed no statistically significant difference between these groups (Figure 5G-H). These findings strongly suggest that TPM4 serves as a robust predictor for LNM in PTC. Additionally, we examined protein expression profiles of both markers using the HPA database, comparing their levels in normal thyroid and PTC tissues. The analysis revealed markedly higher protein expression of TPM4 in PTC tissues compared to LOXL1 (Figure 5I-J), further supporting its potential role as a diagnostic marker.

### Upregulation of TPM4 expression is mediated by hypoxia and promotes lymph node metastasis of PTC through EMT

**Figure 6:**
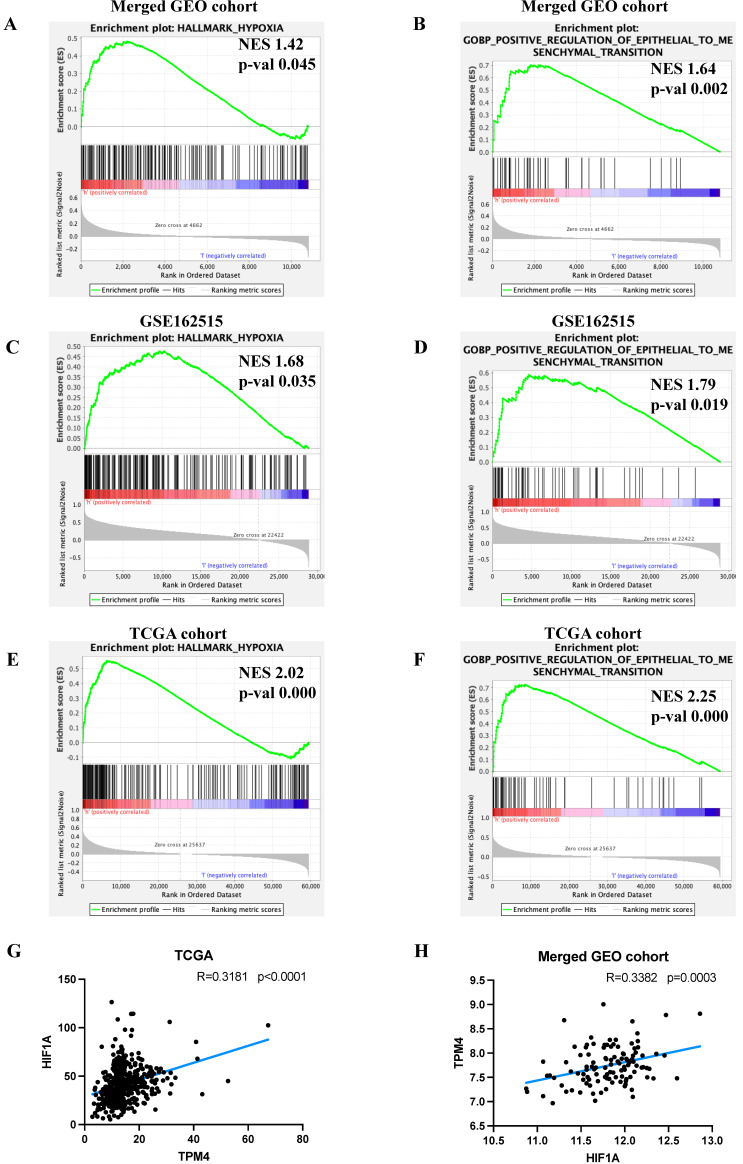
** Exploring the mechanism underlying lymph node metastasis in PTC (A-F)** GSEA analyses between high-expression TPM4 and low-expression TPM4 groups. **(G-H)** Scatter plots illustrating the correlation between TPM4 and HIF1A expression levels.

To elucidate the molecular mechanisms by which TPM4 promotes LNM in PTC, we performed GSEA. Our analysis revealed that the high TPM4 expression group exhibited significant enrichment in both hypoxia and EMT pathways across both GEO cohorts and TCGA cohort (Figure 6A-F). Notably, correlation analyses demonstrated a significant positive association between TPM4 and HIF1A expression in both the TCGA cohort (R_TCGA_= 0.3181, p_TCGA_ < 0.0001) and the merged GEO cohort (R_GEO_ = 0.3382, p_GEO_ = 0.0003) (Figure 6G-H). These findings suggest that hypoxia may upregulate TPM4 expression, which in turn promotes EMT in PTC, ultimately facilitating LNM.

### Overview of single-cell transcriptomic data from PTC with lymph node metastasis

**Figure 7:**
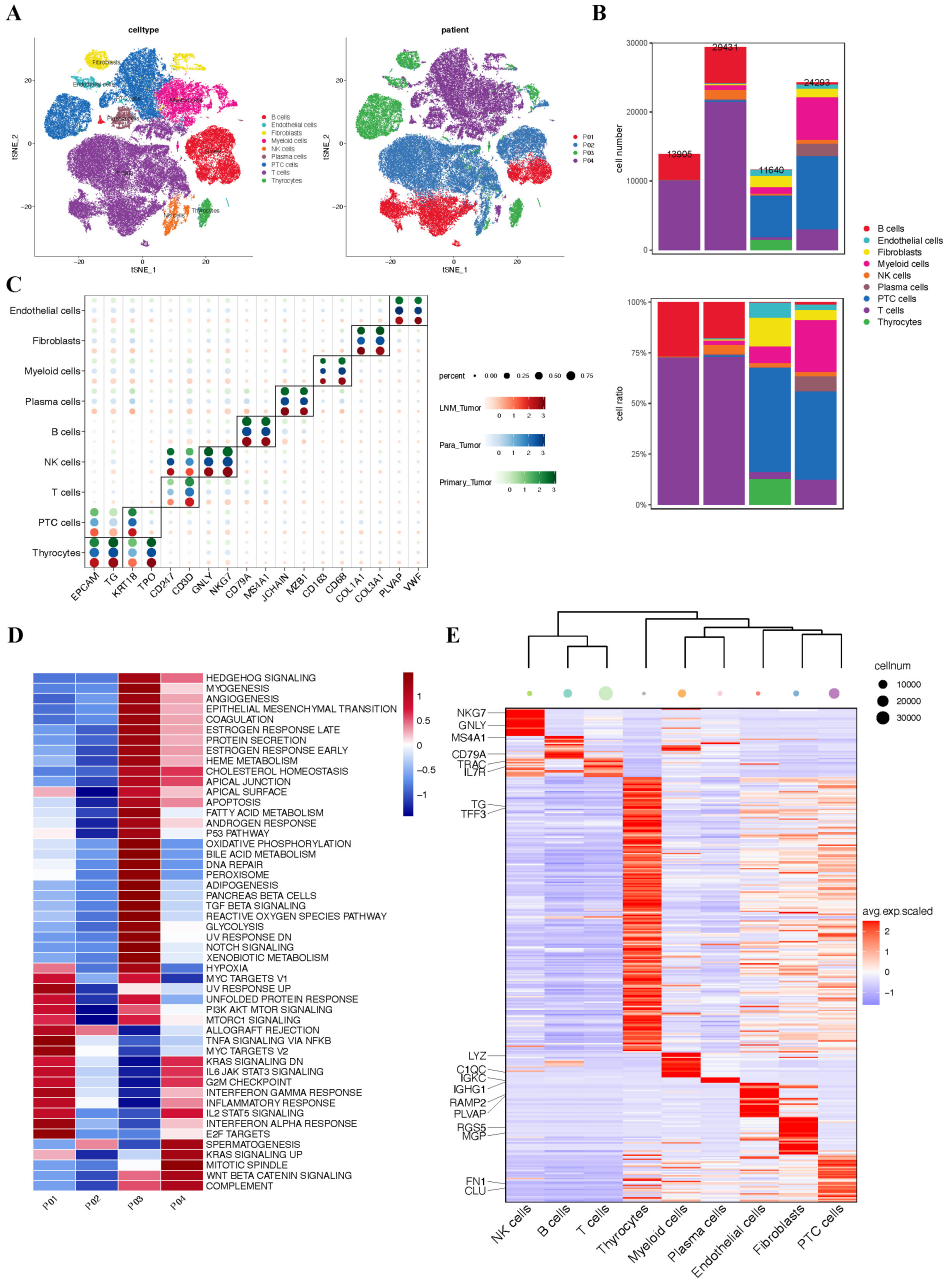
** Single-cell atlas of PTC samples with lymph node metastasis (A)** t-SNE plot of all four patients. **(B)** Quantitative distribution and proportional representation of each cell lineage across the four patients. **(C)** Bubble plot displaying selected marker genes for nine major cell lineages. **(D)** Heatmap of signaling pathway activity scores across four patients, quantified by GSVA. **(E)** Heatmap highlighting the top two characteristic markers for each of the nine principal cell lineages.

Having established through bulk RNA sequencing analysis that hypoxia upregulates TPM4 expression in PTC with LNM, leading to EMT pathway activation, we sought to validate these findings at single-cell resolution. We analyzed the GSE184362 dataset, which comprises single-cell RNA-seq data from four PTC patients, including matched primary tumor and metastatic lymph node samples, along with an adjacent non-tumor sample enriched in normal thyroid epithelial cells. Following rigorous quality control and data preprocessing, we identified 79,269 high-quality cells that clustered into nine distinct cell lineages. These cellular populations were visualized using t-SNE dimensionality reduction (Figure 7A). The distribution of cells across patients and the relative proportion of each cell lineage were presented in a comprehensive bar plot (Figure 7B). Cell lineage-specific marker expression patterns across all samples were illustrated in a bubble plot (Figure 7C). To assess pathway activation states, we performed GSVA to generate pathway enrichment scores for each patient (Figure 7D). Additionally, we constructed a heatmap displaying the top two characteristic markers for each identified cell lineage (Figure 7E).

### Identification of malignant cells and analysis of differential expression gene between PTC cells

**Figure 8:**
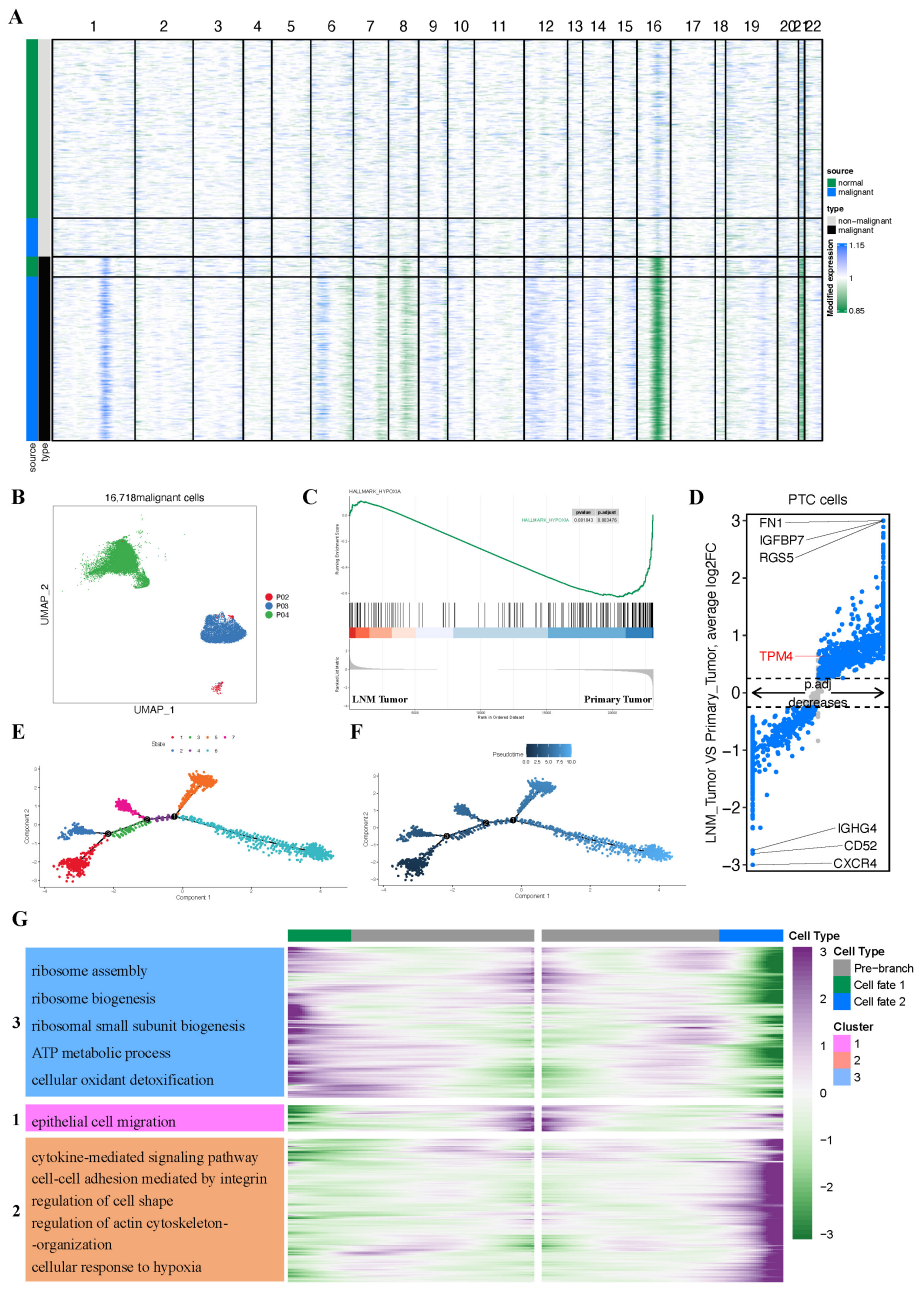
** Malignant cell clusters and common malignant signatures in PTC (A)** Chromosomal landscape of inferred large-scale CNVs distinguishing malignant from non-malignant epithelial cells. **(B)** t-SNE visualization of 16,718 malignant cells derived from 4 patients revealing tumor-specific clustering patterns. **(C)** GSEA comparison between primary tumor and LNM tumor groups. **(D)** Volcano plot highlighting DEGs between primary tumor and LNM tumor groups. **(E)** Trajectory analysis revealing seven distinct cell states within PTC cell. **(F)** Monocle pseudotime trajectory visualization depicting the developmental continuum of PTC cells from initial to terminal states. **(G)** Heatmap of the enrichment analysis between state 5 and state 6.

Since PTC originated from thyroid follicular epithelium, we isolated all epithelial cells based on our initial clustering and lineage identification. To distinguish malignant from non-malignant cells, we inferred chromosome CNVs profiles for each epithelial cell and performed hierarchical clustering of these profiles. Importantly, each of the three primary tumors (P02, P03, and P04) was individually compared with the normal thyroid sample from P03. This analysis identified 261 presumptive non-malignant cells in the P03 primary tumor sample and 135 presumptive malignant cells in the P03 adjacent tumor sample (Figure 8A). Cells exhibiting whole chromosome deletions and amplifications were classified as malignant, yielding a total of 16,718 malignant cells for subsequent analyses (Figure 8B).

GSEA analysis revealed enrichment of the hypoxia pathway in the primary tumor group (Figure 8C). Furthermore, we identified TPM4 as significantly upregulated in the LNM tumor group (adjusted p-value = 3.54e-34, |log2FC| = 0.64) (Figure 8D). These findings suggested that the hypoxic microenvironment in primary tumors may upregulate TPM4 expression in PTC cells, potentially facilitating lymph node metastasis.

We employed pseudotime methods to simulate the differentiation trajectories of 2,307 thyroid cancer cells expressing TPM4. This analysis revealed seven distinct cellular states based on various time points (Figure 8E). According to the pseudotime sequence generated by the Monocle2 algorithm, we designated the branch from state one as the starting point (Figure 8F). Our hypothesis was that cells progressing along the pseudotime trajectory would exhibit increased TPM4 expression accompanied by activation of EMT pathway. Notably, the main trajectory preceding branch 1 showed significant enrichment in the EMT signaling pathway, indicating that tumor cells undergo epithelial-mesenchymal transition during this progression (Figure 8G) (Table S4). To further investigate the molecular changes at the terminal stages, we focused on branch 1 to analyze the signaling pathways enriched in PTC cells at state 6. GO enrichment analysis revealed that cells at this terminal state (state 6) were enriched in multiple pathways, including cytokine-mediated signaling, integrin-mediated cell-cell adhesion, regulation of cell shape, actin cytoskeleton organization, and cellular response to hypoxia, among others (Figure 8G) (Table S4). These enrichment patterns suggest that following EMT activation in the main trajectory, cells reaching state 6 undergo additional changes in cytoskeletal architecture and cellular morphology, potentially accompanied by upregulation of TPM4 expression.

### Progressive evolution of hypoxia and TPM4 expression in PTC cells

**Figure 9:**
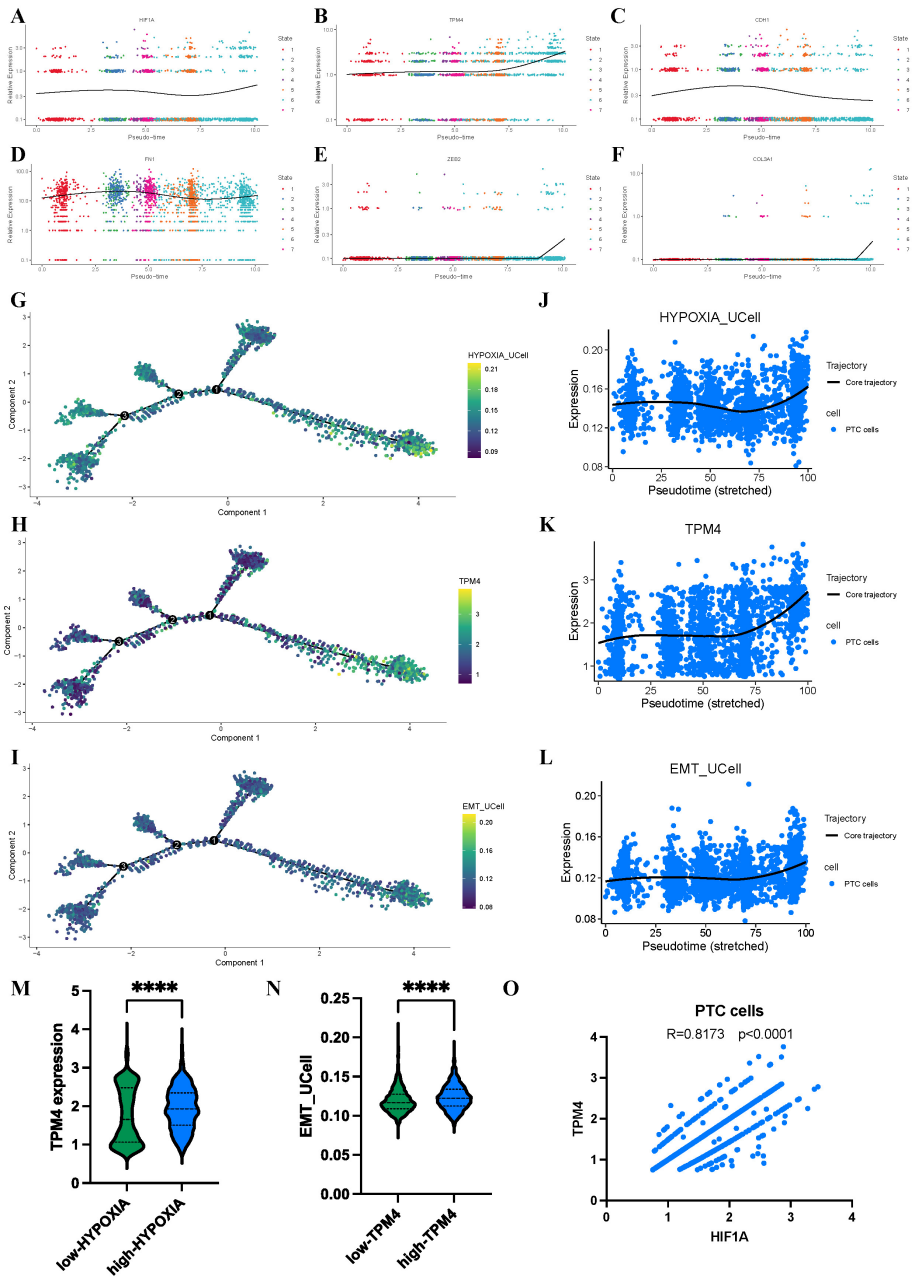
** Dynamic changes in hypoxia pathway, TPM4 expression, and EMT pathway in PTC cells based on scRNA-seq analysis (A-F)** Identification of HIF1A, TPM4, and EMT-related markers throughout cellular evolution. **(G-I)** Single-cell developmental trajectory analysis of TPM4 expression, hypoxia pathway activation, and EMT pathway progression. **(J-L)** Trend analysis of TPM4 expression levels, hypoxia pathway scores, and EMT pathway scores along pseudotime trajectories. **(M)** Comparative analysis of TPM4 expression between low-HYPOXIA and high-HYPOXIA groups. **(N)** Comparative analysis of EMT pathway scores between low-TPM4 and high-TPM4 groups. **(O)** Correlation analysis between HIF1A and TPM4 expression in PTC cells.

To further elucidate the dynamic relationship between hypoxia, TPM4 expression, and EMT pathway activation in PTC cells, we employed pseudotime analysis to model the differentiation trajectories of relevant markers and pathway signatures. Our analysis revealed distinctive temporal patterns as PTC cells progressed along their differentiation trajectory. The HIF1A and TPM4 showed progressive upregulation, alongside established mesenchymal markers FN1, ZEB2, and COL3A1, while the epithelial marker CDH1 exhibited a reciprocal downregulation pattern (Figure 9A-F). To visualize these dynamics comprehensively, we generated trajectory plots illustrating the evolution of TPM4 expression alongside hypoxia pathway and EMT pathway activity during PTC cell differentiation (Figure 9G-I). Notably, all three parameters—TPM4 expression, hypoxia pathway, and EMT pathway—showed coordinated upregulation as cells advanced through differentiation states, suggesting a functional interrelationship between these molecular features (Figure 9J-L). Further statistical analysis confirmed this relationship, with comparative histograms demonstrating significantly elevated TPM4 expression in the high-HYPOXIA group relative to the low-HYPOXIA group. Similarly, EMT pathway activity was markedly enhanced in the high-TPM4 group compared to the low-TPM4 group. Correlation analysis revealed a strong positive association between HIF1A expression and TPM4 levels in PTC cells, further supporting the interconnected nature of hypoxia response and TPM4-mediated EMT processe in thyroid cancer progression.

### Hypoxia upregulates TPM4 to promote cell invasion and migration by regulating EMT in PTC

**Figure 10:**
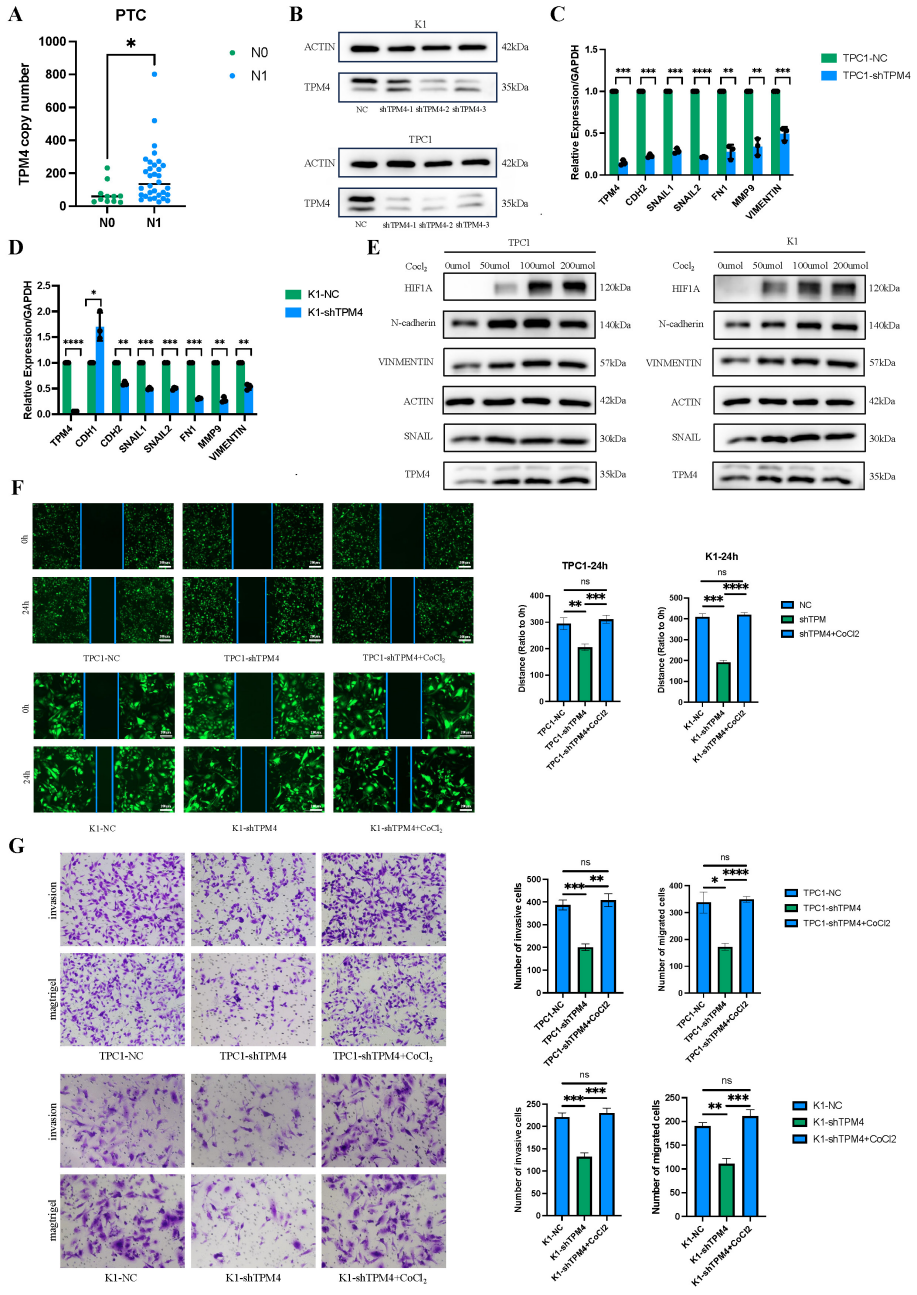
** The hypoxia-TPM4-EMT axis regulates the invasion and migration of PTC cells (A)** RT-qPCR quantification of TPM4 mRNA expression levels in clinical PTC specimens. **(B)** Western blot validation data of TPM4 knockdown efficiency in TPC1 and K1 cell lines. **(C-D)** RT-qPCR analysis of TPM4 and EMT-related marker mRNA expression across shTPM4-TPC1 and shTPM4-K1 cell lines. **(E)** Western blot analysis revealing expression profiles of TPM4 and EMT-related markers in K1 and TPC1 cell lines under hypoxic conditions. **(F)** Wound healing assay evaluating migration capacity of shTPM4-TPC1 and shTPM4-K1 cell lines. **(G)** Transwell assays with and without Matrigel coating to assess invasion and migration capabilities of shTPM4-TPC1 and shTPM4-K1 cell lines, respectively. (* p < 0.05, ** p < 0.01, *** p < 0.001, **** p < 0.0001)

To investigate the role of TPM4 in PTC with LNM, we performed RT-qPCR and Western blot analyses to examine TPM4 mRNA and protein levels in clinical samples and thyroid cancer cell lines. Among 43 clinical PTC samples, TPM4 expression was significantly higher in the lymph node-positive (N1) group compared to the lymph node-negative (N0) group (p < 0.05) (Figure 10A). Chi-square analysis comparing patients with high and low TPM4 expression in tumor samples demonstrated significant differences in lymph node metastasis status (N stage) between the two groups (Table 2). We established stable TPM4 knockdown in TPC1 and K1 cell lines, with shTPM4-2 showing the most efficient knockdown effect (Figure 10B). We then knocked down TPM4 in K1 and TPC1 cells and measured the expression of EMT-related markers. The results showed that TPM4 knockdown effectively reduced the mRNA expression of EMT-related markers in K1 and TPC1 cells (Figure 10C-D). To explore whether hypoxia could upregulate TPM4 expression and affect EMT in PTC cells, we treated cells with CoCl₂ (50, 100, and 200 μmol/L) for 24 hours to induce hypoxia and upregulate HIF1A expression. Western blot analysis revealed that compared to control cells, TPM4, N-cadherin, Vimentin, and Snail expression were elevated in both K1 and TPC1 cells under hypoxic conditions, indicating that hypoxia can induce TPM4 upregulation and subsequently influence EMT (Figure 10E-F). The results of wound healing and transwell assays demonstrated that TPM4 knockdown effectively inhibited migration and invasion of thyroid cancer cells (Figures 10G-H). In conclusion, these findings suggest that the hypoxia-TPM4-EMT axis may play a crucial role in the biological process of lymph node metastasis in PTC.

## Discussion

PTC exhibits high invasiveness and a significant propensity for LNM, with statistical data indicating lymph node recurrence rates reaching up to 74%^33^. Recurrent tumors pose greater therapeutic challenges, often necessitating reoperation, which carries elevated surgical risks and potentially compromises patients' quality of life^34^. Among various risk factors, intratumoral hypoxia stands out as a crucial driver of cancer cell metastasis^35^, ^36^. Therefore, elucidating the molecular mechanisms underlying hypoxia-driven metastasis remains imperative. To our knowledge, this study represents the first demonstration that hypoxia promotes PTC lymph node metastasis through TPM4 upregulation.

Bulk-seq technology has emerged as a powerful tool for investigating gene functions, disease mechanisms, and biological processes in both medical research and bioinformatics. Its applications span both tumor and non-tumor contexts^37^, ^38^. Single-cell analysis enables high-resolution identification of gene expression patterns and dynamic changes at the cellular level, proving particularly valuable for understanding cellular responses and transitions during biological processes. The reliability of single-cell RNA sequencing in exploring gene-specific biological characteristics has been extensively documented^39^, ^40^. In this study, we integrated these complementary sequencing approaches to enhance the robustness and precision of our identification of LNM-related biomarkers in PTC.

To identify novel LNM-associated biomarkers in PTC, we employed a screening strategy based on two phenotypes linked to thyroid cancer dedifferentiation. Initial analyses of TCGA RNA-seq data using WGCNA, LASSO, and ML approaches identified two candidate genes - TPM4 and LOXL1 - strongly associated with PTC lymph node metastasis. Subsequent validation across multiple GEO datasets revealed that only TPM4 showed consistently elevated expression in the lymph node metastasis group with statistical significance. Further analysis using the HPA database demonstrated robust TPM4 expression in PTC tissues, while LOXL1 expression was negligible. Tropomyosin serves as a key regulator of actin filaments, influencing stress fiber organization, lamellipodia and lamellae formation, and cell migration velocity^41^-^43^. TPM4, a tropomyosin protein predominantly expressed in non-muscle cells, has been documented to show increased expression across various malignancies^44^-^46^. Given the absence of prior studies examining TPM4 expression and function in thyroid cancer, we undertook this investigation.

WGCNA analysis revealed TPM4 among the hub genes in the most significant hypoxia-associated module, suggesting a strong correlation between hypoxia and TPM4. GSEA analysis further supported hypoxia as a potential inducer of TPM4 upregulation, promoting EMT in PTC. To further elucidate the interplay between hypoxia pathway, TPM4 expression, and EMT pathway, we conducted pseudotime analysis using scRNA-seq data from PTC cells with LNM. Our results demonstrated that as tumor cells progress through differentiation, hypoxia pathway enrichment coincides with TPM4 upregulation and EMT pathway activation. Moreover, PTC cells exhibiting high TPM4 expression showed concurrent elevation in both hypoxia and EMT pathway signatures. These findings strongly suggest that the hypoxia-TPM4-EMT axis serves as a critical driver of PTC with LNM.

Previous research has demonstrated that elevated TPM4 expression promotes cancer cell migration and invasion in various malignancies, including glioma, breast cancer, and lung cancer, through F-actin stabilization^19^, ^46^, ^47^. Harris et al. demonstrated that HIF-1α modulates RIOK3 expression, and RIOK3 depletion reduces TPM3-F-actin binding, consequently diminishing F-actin stability and inhibiting breast cancer cell invasion and migration^19^. While our findings indicate that the hypoxia pathway upregulates TPM4 expression, we propose that HIF-1α, a master regulator of hypoxic response, may not directly regulate TPM4. Instead, HIF-1α might transcriptionally regulate specific kinases that influence TPM4-F-actin binding, thereby promoting tumor cell motility. Further investigation is warranted to elucidate the precise mechanisms by which HIF-1α influences TPM4 expression.

Our study made several novel contributions: we first demonstrated that hypoxia-induced TPM4 upregulation in PTC influences tumor cell invasion and migration, facilitating lymph node metastasis. Additionally, we pioneered the investigation of TPM4 role in PTC, supporting our findings through comprehensive bioinformatic analyses and preliminary experimental validation. However, certain limitations warrant attention in future research. First, deeper mechanistic investigation of the hypoxia-TPM4 axis in tumor cell metastasis is needed. Second, while our algorithmic approach addressed batch effects, the limited sample size in our single-cell analysis suggests that additional samples would be valuable to minimize the impact of individual variation on our findings.

## Conclusion

Through an integrative multi-omics approach combining comprehensive bioinformatics analyses of TCGA and GEO databases at both bulk-seq and single-cell RNA-seq levels, along with rigorous experimental validation, we systematically identified and validated TPM4 as a key hypoxia-responsive gene in PTC progression. Our study elucidates a novel mechanism whereby the hypoxic microenvironment in papillary thyroid cancer drives TPM4 upregulation, subsequently inducing epithelial-mesenchymal transition in tumor cells and promoting lymph node metastasis. These findings provide a potential biomarker for risk stratification of PTC patients and offer new insights into the pathogenesis of lymph node metastasis, potentially serving as a promising therapeutic target for preventing metastatic progression in clinical management.

## Supplementary Materials

Figure S1: Heatmap depicting FSTL1 expression patterns and clinicopathological characteristics; Table S1: HARRIS_HYPOXIA gene set and REACTOME_AUTOPHAGY gene set; Table S2: Hypoxia phenotype score and autophagy phenotype score of each PTC sample in the TCGA-THCA dataset; Table S3: The qPCR primer sequences of EMT-related markers and the shTPM4 sequences; Table S4: Enrichment analysis of differentially expressed genes in single-cell pseudotime analysis.

## Figures and Tables

**Table 1:** Comparison of clinicopathological features of PTC patients between high- and low-TPM4 expression groups in TCGA dataset.

Clinicopathological features	Low-TPM4 group (%)	High-TPM4 group (%)	χ^2^	p
Gender			0.8856	0.3467
Male	61 (24.60)	71 (28.51)		
Female	187 (75.40)	178 (71.49)		
Age (Year)			5.2398	0.0221
< 60	182 (73.39)	197 (79.12)		
≥ 60	66 (26.61)	52 (20.88)		
T			2.5196	0.4718
T1	77 (31.05)	66 (26.51)		
T2	84 (33.87)	80 (32.13)		
T3	77 (31.05)	88 (35.34)		
T4	9 (03.63)	14 (05.62)		
T unknow	1 (00.40)	1 (00.40)		
N			21.5818	3.39e-06
N0	133 (53.63)	140 (56.23)		
N1	81 (32.66)	94 (37.75)		
N unknow	34 (13.71)	15 (06.02)		
M			3.3909	1
M0	132 (53.23)	146 (58.63)		
M1	4 (01.61)	4 (01.61)		
M unknow	112 (45.16)	99 (39.76)		
Stage			12.6623	0.0268
Stage I	131 (52.82)	150 (60.24)		
Stage II	35 (14.11)	16 (06.43)		
Stage III	57 (22.98)	53 (21.28)		
Stage IV	23 (09.27)	30 (12.05)		
Stage unknow	2 (00.82)	0 (00.00)		
Status			0.0688	0.7931
Alive	241 (97.18)	240 (96.39)		
Dead	7 (02.82)	9 (03.61)		

**Table 2:** Comparison of clinicopathological features of PTC patients between high- and low-TPM4 expression groups in clinical specimens

Clinicopathological features	Low-TPM4 group (%)	High-TPM4 group (%)	χ^2^	p
Gender			0.0112	0.9156
Male	7 (33.33)	7 (31.82)		
Female	14 (66.67)	15 (68.18)		
Age (Year)			Fisher	>0.9999
<60	18 (85.71)	19 (86.36)		
≥60	3 (14.29)	3 (13.64)		
T			3.1780	0.3649
T1	15 (71.43)	10 (45.45)		
T2	3 (14.29)	6 (27.27)		
T3	1 (04.76)	3 (13.64)		
T4	2 (09.52)	3 (13.64)		
N			Fisher	0.0339
N0	9 (42.86)	2 (09.09)		
N1	13 (57.14)	20 (90.91)		
Stage			2.6040	0.2720
Stage I	19 (90.48)	16 (72.73)		
Stage II	1 (04.76)	4 (18.18)		
Stage III	1 (04.76)	2 (09.09)		
Tumor site			1.3920	0.4986
Right lobe	4 (19.05)	7 (31.82)		
Left lobe	7 (33.33)	8 (36.36)		
Bilateral lobes	10 (47.62)	7 (31.82)		
Tumor lesion			1.1330	0.2871
Single lesion	9 (42.86)	13 (59.09)		
Multiple lesions	12 (57.14)	9 (40.91)		

## Abbreviations

PTC: Papillary thyroid cancer
LNM: Lymph node metastasis
EMT: Epithelial-mesenchymal transition
DTC: Differentiated thyroid cancer
TCGA: The cancer genome atlas
GEO: Gene expression omnibus
ssGSEA: Single sample gene set enrichment analysis
WGCNA: Weighted gene co-expression network analysis
GS: Gene significance
MM: Module membership
LASSO: Least absolute shrinkage and selection operator
GO: Gene ontology
BP: Biological process
MF: Molecular function
CC: Cellular component
KEGG: Kyoto encyclopedia of genes and genomes
GSVA: Gene set variation analysis
ML: Machine learning
XGB: Extreme gradient boosting
RF: Random forest
GLM: Generalized linear model
SVM: Support vector machine
RMSE: Root mean square error
ROC: Receiver operating characteristic
AUC: Area under curve
HPA: Human protein atlas
GEPIA 2: Gene expression profiling interactive analysis 2
GSEA: Gene set enrichment analysis
scRNA-seq: Single-cell RNA sequencing
CNVs: Copy number variations
ACT: Annotation of cell types
tSNE: T-distributed stochastic neighbor embedding
Umap: Uniform manifold approximation and projection
DEGs: Differentially expressed genes
RT-qPCR: Real-time quantitative polymerase chain reaction
TDS: Thyroid differentiation score
