# Supplementary material for: Hypoxia upregulate TPM4 expression to strengthen epithelial-mesenchymal transition that promotes lymph node metastasis of papillary thyroid cancer
Source: J Cancer. 2025 Jul 11;16(10):3216–34. doi: 10.7150/jca.116524 (PMC12305566; doi:10.7150/jca.116524)
Supplement: Supplementary file 1 — Figure S1: Heatmap depicting FSTL1 expression patterns and clinicopathological characteristics; Table S1: HARRIS_HYPOXIA gene set and REACTOME_AUTOPHAGY gene set; Table S2: Hypoxia phenotype score and autophagy phenotype score of each PTC sample in the TCGA-THCA dataset; Table S3: The qPCR primer sequences of EMT-related markers and the shTPM4 sequences; Table S4: Enrichment analysis of differentially expressed genes in single-cell pseudotime analysis. [file jcav16p3216s1.zip › Supplemental Text and Figures/Supplementary materials(Figure S1 Table S3).pdf]

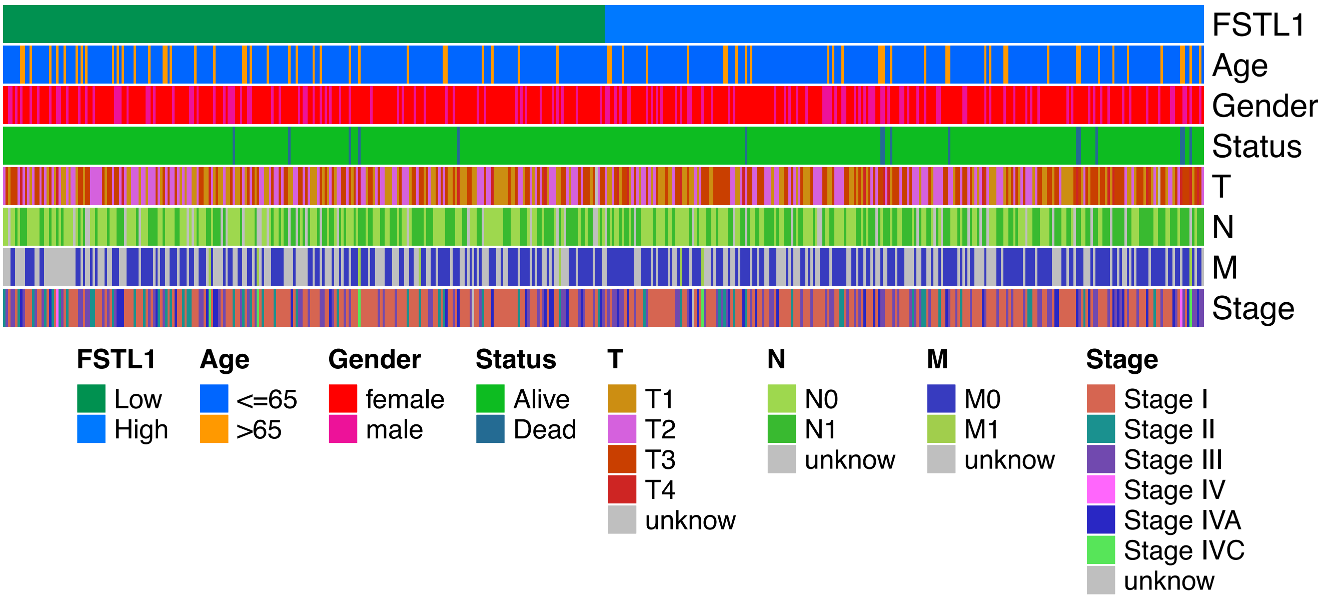
FSTL1
Age
Gender
Status
T
N
M
Stage
FSTL1
Low
High
Age
<=65
>65
Gender
female
male
Status
Alive
Dead
T
T1
T2
T3
T4
unknow
N
N0
N1
unknow
M
M0
M1
unknow
Stage
Stage I
Stage II
Stage III
Stage IV
Stage IVA
Stage IVC
unknow

Table S3

RT-qPCR Primer

Gene	Sense (5'-3')
TPM4-1F	AGCTGACGCACCTCCAGAAGAA
TPM4-1R	CCTGTCCAACTCCTCCTCAACGA
HIF1A-F	AGTTCCGCAAGCCCTGAAAGC
HIF1R-R	TCATCAGTGGTGGCAGTGGTAGT
CDH2-F	TCCTGCTTATCCTTGTGCTGATGT
CDH2-R	TGGTCTTCTTCTCCTCCACCTTCT
MMP9-F	ACCTCGAACTTTGACAGCGAC
MMP9-R	GAGGAATGATCTAAGCCCAGC
SNAI1-F	TCGGAAGCCTAACTACAGCGA
SNAI1-R	AGATGAGCATTGGCAGCGAG
SNAI2-F	TGCCTGTCATACCACAACCAGAGAT
SNAI2-R	TGGAGGAGGTGTCAGATGGAGGA
VIM-F	ACACCCTGCAATCTTTCAGACA
VIM-R	GATTCCACTTTGCGTTCAAGGT
FN1-F	CCTTCATGGCAGCGGTTT
FN1-R	AGCGTCCTAAAGACTCCATGATCT

shRNA target sequence

Gene	Sense (5'-3')
TPM4-sh-1	CCCAGTATCTAGTCGTGGATA
TPM4-sh-2	CTGGAAGAAGAACTCAAGAAT
TPM4-sh-3	CCTGGAAGAAGAACTCAAGAA
